# Case report: A novel mutation of glial fibrillary acidic protein gene causing juvenile-onset Alexander disease

**DOI:** 10.3389/fneur.2024.1362013

**Published:** 2024-03-20

**Authors:** Carmela Romano, Emanuele Morena, Simona Petrucci, Selene Diamant, Martina Marconi, Lorena Travaglini, Ginevra Zanni, Maria Piane, Marco Salvetti, Silvia Romano, Giovanni Ristori

**Affiliations:** ^1^Department of Human Neurosciences, Sapienza University of Rome, Sant’Andrea Hospital, Rome, Italy; ^2^Department of Neurosciences, Mental Health and Sensory Organs, Sapienza University of Rome, Sant’Andrea Hospital, Rome, Italy; ^3^Department of Clinical and Molecular Medicine, Faculty of Medicine and Psychology, Sapienza University of Rome, Rome, Italy; ^4^S. Andrea University Hospital, Rome, Italy; ^5^Laboratory of Medical Genetics, Bambino Gesù Children’s Hospital, IRCCS, Rome, Italy; ^6^Genetics and Rare Diseases Research Division, Unit of Neuromuscular and Neurodegenerative Disorders, Bambino Gesù Children’s Hospital, IRCCS, Rome, Italy; ^7^IRCCS Istituto Neurologico Mediterraneo (INM) Neuromed, Pozzilli, Italy; ^8^Neuroimmunology Unit, IRCCS Fondazione Santa Lucia, Rome, Italy

**Keywords:** Alexander disease, leukodystrophy, GFAP mutation, astrocytes, ataxia

## Abstract

Alexander disease (AxD) is a rare inherited autosomal dominant (AD) disease with different clinical phenotypes according to the age of onset. It is caused by mutations in the glial fibrillary acid protein (GFAP) gene, which causes GFAP accumulation in astrocytes. A wide spectrum of mutations has been described. For some variants, genotype–phenotype correlations have been described, although variable expressivity has also been reported in late-onset cases among members of the same family. We present the case of a 19-year-old girl who developed gait ataxia and subtle involuntary movements, preceded by a history of enuresis and severe scoliosis. Her mother has been affected by ataxia since her childhood, which was then complicated by pyramidal signs and heavily worsened through the years. Beyond her mother, no other known relatives suffered from neurologic syndromes. The scenario was further complicated by a complex brain and spinal cord magnetic resonance imaging (MRI) pattern in both mother and daughter. However, the similar clinical phenotype made an inherited cause highly probable. Both AD and autosomal recessive (AR) ataxic syndromes were considered, lacking a part of the proband’s pedigree, but no causative genetic alterations were found. Considering the strong suspicion for an inherited condition, we performed clinical exome sequencing (CES), which analyzes more than 4,500 genes associated with diseases. CES evidenced the new heterozygous missense variant c.260 T > A in exon 1 of the glial fibrillary acidic protein (GFAP) gene (NM_002055.4), which causes the valine to aspartate amino acid substitution at codon 87 (p. Val87Asp) in the GFAP. The same heterozygous variant was detected in her mother. This mutation has never been described before in the literature. This case should raise awareness for this rare and under-recognized disease in juvenile–adult cases.

## 1 Introduction

Alexander disease (AxD) is a very rare inherited leukodystrophy with a progressive course. Its prevalence is not well known, and the only population-based prevalence estimate is one in 2.7 million (1). AxD is caused by mutations in the *glial fibrillary acid protein (GFAP) gene*, located in chromosome 17q21, which encodes a type III intermediate filament protein that is predominantly expressed in astrocytes. The most reported variants are point mutations at exon 1 (54%), exon 4 (31%), and exons 8, 6, and 5 (7, 4, and 3%, respectively). The *GFAP* variants act as gain-of-function mutations that break up the dimerization of astrocytes’ intermediate filaments, causing abnormal protein aggregation, called Rosenthal fibers, in the astrocyte cytoplasm (2). The Rosenthal fibers, which are aggregates of GFAP, heat shock protein 27 (HSP27), and alpha B-crystallin, are the pathological hallmark of the disease (3). The accumulation of Rosenthal fibers causes astrocyte dysfunction (4). However, other mechanisms, apart from protein aggregation, seem to be responsible for AxD pathology. It seems in fact that GFAP mutations cause proteasome activity inhibition, chemokine, and nitric oxide production, and consequently, oxidative stress, activation of cell stress patterns, and changes in astrocyte morphology. Furthermore, these mutant astrocytes promote an inflammatory environment in the CNS (5). Whatever the cause, the damage begins in the astrocytes and then extends beyond involving other cellular elements through probably microglial activation (2). All these changes are responsible for white matter degeneration and neuronal loss (5).

AxD usually has well-defined clinical characteristics in infants and children, but it can also be present in adults with non-specific neurological manifestations. Based on these different age-related clinical spectrums, four clinical groups were distinguished: neonatal form, infantile form, juvenile form, and adult form (2, 6). The neonatal form is characterized by neurodevelopmental delay and regression, seizures, and gastrointestinal manifestations with a rapid progression to death within 2 years; the infantile form presents with variable developmental issues, seizures, ataxia, hyperreflexia, spasticity, hydrocephalus, and megalencephaly; the juvenile form is characterized by mild developmental delay, bulbar signs, vomiting, scoliosis, autonomic dysfunction, spasticity, ataxia, and epilepsy; and the adult form presents with bulbar or pseudobulbar findings (palatal myoclonus, dysphagia, dysphonia, dysarthria, or slurred speech), motor/gait abnormalities with pyramidal tract signs, or cerebellar abnormalities (2, 6). In 2001, Van der Knaap et al. identified, in a population of infantile-onset AxD, five magnetic resonance imaging (MRI) criteria to diagnose AxD: 1. extensive cerebral white matter abnormalities with a frontal preponderance; 2. periventricular rim of decreased signal intensity on T2-weighted images and elevated signal intensity on T1-weighted images; 3. abnormalities of the basal ganglia and thalami, such as swelling or atrophy; 4. brain stem abnormalities, particularly involving the medulla and midbrain; 5. contrast enhancement of one or more of the following: ventricular lining (*Garland-like feature*), periventricular rim, frontal white matter, optic chiasm, fornix, basal ganglia, thalamus, dentate nucleus, and brain stem (7).

Furthermore, several MRI studies in AxD showed significant differences in radiological presentation between infant and juvenile/adult-onset, with a predominant atrophy and signal alterations in the brainstem and upper spinal cord in juvenile/adult-onset, as well as fewer supratentorial periventricular white matter abnormalities in infantile-onset (8).

In 2011, a new classification was proposed based on the distribution of lesions and the age of onset (9), dividing AxD into two types: type I, characterized by frontal lobe distribution and infantile-onset; type II, characterized by hindbrain predominance of lesions and a more variable age of onset (juvenile and adult). To date, several case reports have described the most common MRI alterations found in type II AxD: 1. significant atrophy of the medulla and spinal cord; 2. involvement of the dentate with less frequent coexisting cerebellar white matter change (10); 3. poor involvement of white matter compared to type I AxD; 4. pial FLAIR signal abnormality, most often concentrated in the medulla; 5. enhancement of middle cerebellar peduncle and brainstem; 6. bilateral involvement of middle cerebellar peduncle, which is a non-specific MRI feature of AxD but is present in several other neurodegenerative diseases (multiple system atrophy, spinocerebellar ataxia, Wilson disease (WD), etc.); 7. “Tadpole atrophy,” consisting of atrophy of the medulla and cervical spinal cord with a relative sparing of the pons; 8. medullary signal change (myelopathy is a common clinical presentation in these patients) associated with spinal atrophy (also enhancement may be present), which leads to a strong suspicion of AxD (8, 11).

The diagnosis of AxD may be suspected in patients with suggestive clinical and radiological features, but it requires genetic confirmation. Nowadays, the use of next-generation sequencing (NGS) technologies is increasing the diagnostic rate.

We report the case of a young woman who developed ataxia, involuntary movements, enuresis, and scoliosis as her main symptoms and signs. Her mother had a similar clinical pattern. The set of clinical, radiological, and anamnestic data leads us to suspect a genetic cause. The clinical exome sequencing (CES) evidenced a new heterozygous missense variant c.260T > A in exon 1 of the *GFAP* gene (NM_002055.4) that causes the valine to aspartate amino acid substitution at codon 87 (p. Val87Asp) in the GFAP both in our patients and in her mother. This mutation has never been described before in the literature.

## 2 Case presentation

A 19-year-old girl (III:1, Figure 1) came to our neurological clinic for the recent appearance of mild imbalance and involuntary movements in her face and upper limbs.

**Figure 1:**
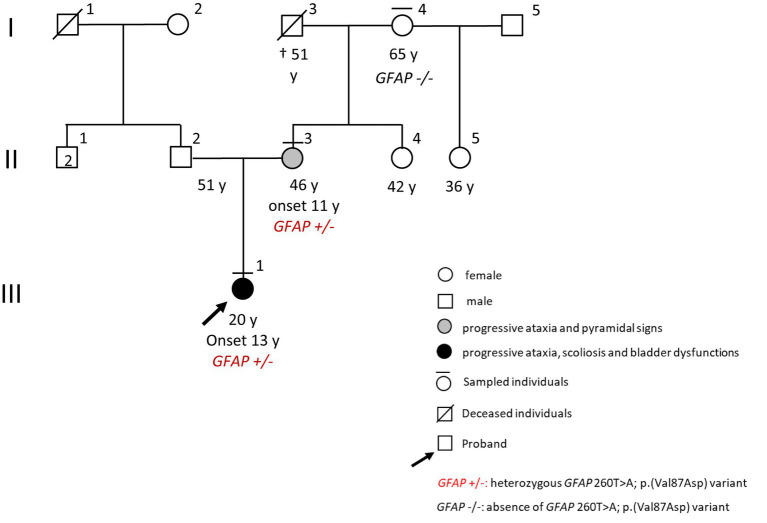
Pedigree.

The patient reported, in her medical history, scoliosis, and episodes of enuresis since she was 13 years old. Moreover, her aunt reported some episodes characterized by lack of attention, in which the patient seemed “lost in her thoughts,” but gave a prompt response when asked. She had normal psychomotor development, and she succeeded in graduating from high school despite some learning difficulties.

Exploring the familiar history, we discovered that her mother (II:3, Figure 1) had a previous diagnosis of primary progressive multiple sclerosis (PPMS) with an unclear clinical course. The first symptom experienced by the mother since her childhood seemed to be ataxia; however, through the years, weakness and stiffness in both legs became the leading symptoms, with a rapid worsening until bedding and percutaneous endoscopic gastrostomy (PEG) feeding when she was 40 years old. Because of her critical clinical condition, we were not able to perform a neurological examination for the mother. However, the last neurological examination performed some years ago in another clinic evidenced spastic paraparesis, slurred speech, and dysphagia. No maternal relatives were referred to have neurologic diseases except for the mother, and no information was available about the father or the paternal branch (Figure 1).

The neurological examination of our patient mainly evidenced difficulties in balance and walking, with significant difficulty performing tandem test walking. Moreover, we evidenced a clear nystagmus in the horizontal gaze bilaterally, a clonus at the ankles with a plantar cutaneous reflex in extension bilaterally, and sporadic dystonic movements in her face and upper limbs.

Therefore, our patient’s presentation with a complex syndrome with prominent cerebellar ataxia, which was phenotypically similar to that of her mother, made an inherited cause highly probable. Nonetheless, we also investigated the possible causes of acquired ataxias, such as vitamin deficiency, autoimmune, and viral causes.

The proband’s pedigree was partially informative; therefore, both autosomal dominant (AD) and autosomal recessive (AR) ataxic syndromes were considered consanguinity, and/or pseudo-dominant inheritance could not be excluded.

We first assessed the most common AD cerebellar ataxias, but the repeat expansion testing excluded any pathogenic amplification in the *ATXN*1, *ATXN2*, *ATXN3*, *CACNA1A*, *ATXN7*, *PPP2R2B*, and *TBP* genes, and no damaging variants in the *KCNC3* and *FXN* genes were detected. Therefore, we performed a NGS analysis for the ataxia-related genes. However, we found no causative genetic alterations.

**Figure 2:**
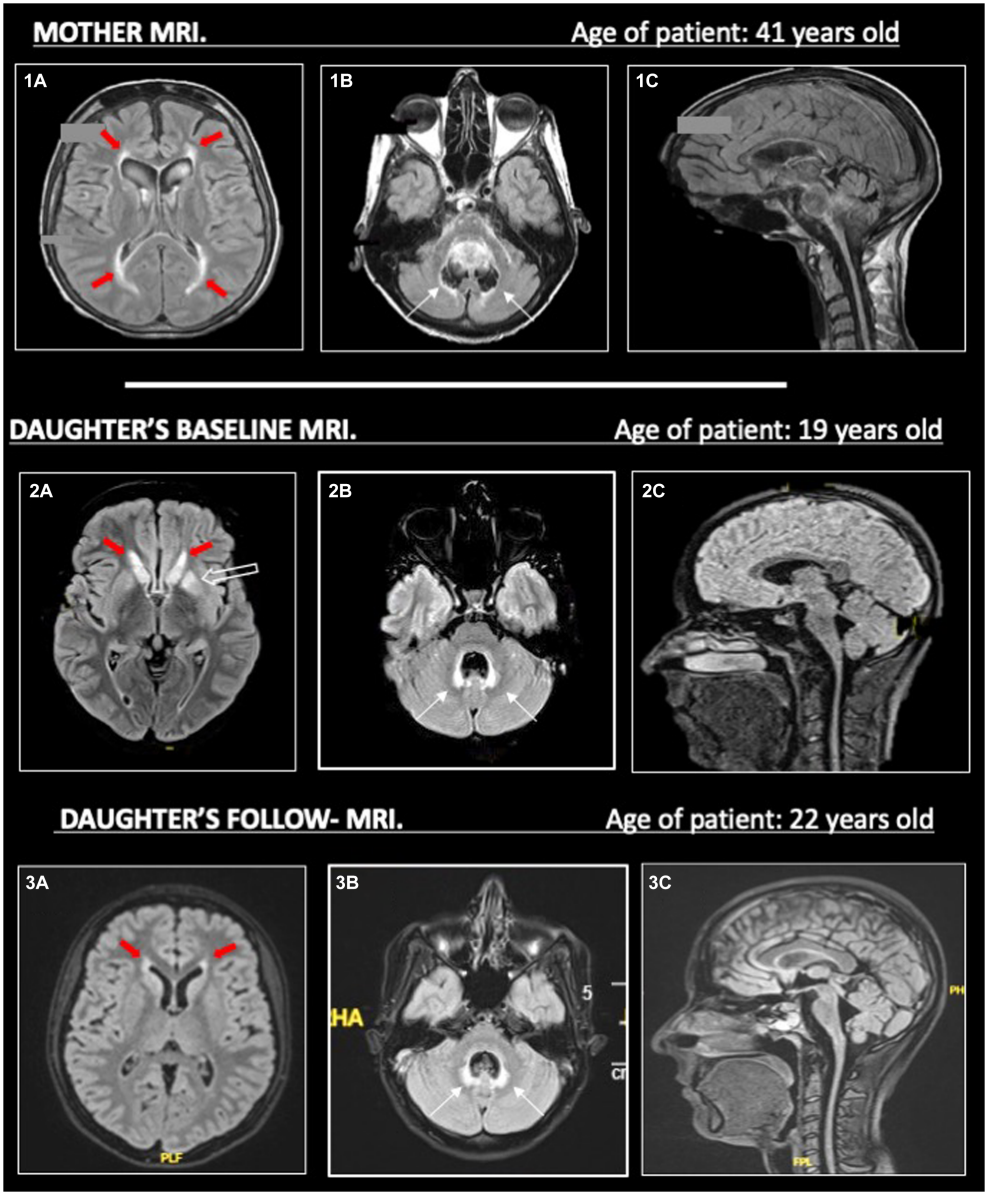
On the left (1a, 2a, 3a), we show T2-hyperintense symmetric periventricular lesions (red-filled arrows) in both mother and daughter. In (2a), we can see T2-hyperintensities in the basal ganglia (empty arrow) of the daughter, less evident in her MRI follow-up in 2023 (3a). On the central column (1b, 2b, 3b), widespread atrophy of the cerebellar vermis is evident in both mother and daughter (1b, 2b), without evident worsening in the follow-up MRI (3b) in the daughter. T2-hyperintensities around the IV ventricle (thin black arrows) are more pronounced in the daughter than the mother, maybe due to a higher cerebellar vermis atrophy. On the right (1c, 2c, 3c), in the sagittal images, we can see prominent atrophy of the cerebellum, brainstem (with spare of pons) and cervical spinal cord. All MRI sequences are FLAIR, apart from (1c) (T1).

In the meantime, the patient performed further exams, including a brain and spinal cord MRI. The MRI evidenced limited T2-hyperintensities involving the periventricular and temporal insular white matter in both hemispheres, and, more interestingly, the T2-hyperintensities also involved basal ganglia, middle cerebellar peduncles, and the area around the IV ventricle; no enhancement was identified in these areas. Moreover, it showed cerebellar vermal and spinal cord atrophy (Figure 2).

Based on the basal ganglia alterations at MRI, as well as on the clinical evidence of cerebellar involvement and dystonia, we performed a ceruloplasmin and copper level dosage and a molecular analysis of the *ATP7B* gene to investigate WD, a rare AR disorder of copper metabolism. However, even these tests resulted in a negative result.

In addition, the patient performed uroflowmetry, which showed neurogenic bladder disease; neuropsychological tests, which evidenced some difficulties with planning skills and episodic memory; and a VideoEEG during sleep, which showed not-induced, rare epileptic spike-and-wave discharges that spread over all of the brain with no clinical correlations.

At this point, we obtained a mother’s MRI, performed at the age of 41 years old. This MRI evidenced white matter abnormalities around the IV ventricle, more limited white matter abnormalities in the supratentorial periventricular area, and pial T2-hyperintensities were described in the brainstem and cerebellum; a severe atrophy of the brainstem, cerebellum (especially the vermis), and cervical spinal cord was described (Figure 2). However, the mother’s MRI accounted for the advanced phase of the disease, and no previous imaging was available.

Even if the MRI patterns of our patient and her mother did not overlap entirely, despite the comparison being made between MRIs performed at different ages and stages of the disease, there was a strong suspicion for an inherited neurodegenerative condition, including leukodystrophies (12).

Therefore, we decided to perform CES, a diagnostic targeted sequencing test that analyzes more than 4,500 genes associated with diseases, by NGS (TruSightONE Expanded Sequencing Kit, Illumina).

The CES evidenced the new heterozygous missense variant c.260 T > A in exon 1 of *GFAP* (NM_002055.4), which causes the valine to aspartate amino acid substitution at codon 87 (p. Val87Asp) in the glial fibrillary acidic protein. No further damaging variants were found in the other analyzed genes. The *GFAP* variant was confirmed in the proband and investigated in the mother (II:3, Figure 1) and in the grandmother (I:4, Figure 1) by Sanger sequencing. This analysis identified the substitution also in the mother, but not in the healthy grandmother (Figure 1, pedigree). Damaging variants in *GFAP* (MIM * 1377 80, chromosome) are responsible for Alexander’s disease (AxD, MIM # 203450), a rare AD neurodegenerative disorder caused by glial fibrillary acidic protein accumulation in astrocytes.

## 3 Discussion

AxD is a very rare inherited leukodystrophy with a progressive and fatal clinical course due to a mutation in the *GFAP gene*, which encodes an intermediate filament protein that is predominantly expressed in astrocytes (2).

AxD clinical presentation is heterogeneous, and different members of the same family may have variable phenotypes. This clinical heterogeneity depends on different variants of the mutation and on different ages of onset (6).

In this case report, ataxia was the leading symptom in the proband and the first clinical manifestation in her mother, which progressively worsened through the years. The management of inherited ataxias may be challenging for clinicians due to the wide clinical phenotypes and the continuous discovery of new genetic mutations. Clinical findings are not sufficient for the diagnosis, and, in the absence of a clear familiar history, misdiagnosis is quite common. In our case, for example, the mother was previously misdiagnosed with PPMS.

The diagnostic process was further complicated by the incomplete knowledge of the patient’s pedigree and the complex neuroimaging patterns in the patient and her mother, taken in different stages of the disease. As illustrated in Figure 2, the predominant atrophy of the brainstem with a spare of pons (tadpole atrophy), the atrophy of the cerebellum and spinal cord, the pial and sub-pial alterations of the brainstem, and the few white matter abnormalities may have suggested the possibility of a type II AxD.

In the end, the CES analysis evidenced the new heterozygous missense variant c.260 T > A in exon 1 of the *GFAP* gene (NM_002055.4). According to the American College of Medical Genetics and Genomic Guidelines, this variant can be classified as likely pathogenic (13). Indeed, i) the c.260T > A in *GFAP* we found in the proband and her mother has never been described to date, either in general population or AxD cases (pathogenic moderate criteria 2, PM2); ii) it is in a mutational hot spot domain of the protein (pathogenic moderate criteria 1, PM1), iii) it is considered pathogenic by the most relevant pathogenicity scores (MetaLR 0.94; Revel 0.93) (pathogenic supporting criteria 3, (PP3); iv) different amino acidic changes at the same codon (p. Val87Ile; p.Val87Leu; p.Val87Gly) were previously described in late-onset AxD patients (pathogenic moderate criteria 5, PM5) (8, 14–16).

These data (PM1, PM2, PM5, PP1, PP3 PP4), together with the maternal segregation and the clinical features of the two women compatible with *GFAP*-related phenotypes (pathogenic supporting 1, PP1, and pathogenic supporting criteria 4, PP4), are in favor of a damaging role of the p.Val87Asp substitution in determining the disease (13).

Currently, our patient is receiving symptomatic treatments such as physical therapy and anti-epileptic drugs (AEDs), and she has overall clinical stability. To date, unfortunately, there are no disease-modifying treatments available for AxD, even if a Phase 1–3 multi-center trial testing antisense oligonucleotides is now ongoing (17).

This clinical case expands the known variable genotype–phenotype correlation in AxD, reporting a new variant causing juvenile AxD, and it should raise awareness for this rare and under-recognized disease in juvenile–adult cases.

## Data availability statement

The datasets presented in this article are not readily available because of ethical and privacy restrictions. Requests to access the datasets should be directed to the corresponding author.

## Ethics statement

Ethical review and approval was not required for the study on human participants in accordance with the local legislation and institutional requirements. Written informed consent from the patients/participants or patients/participants’ legal guardian/next of kin was not required to participate in this study in accordance with the national legislation and the institutional requirements. Written informed consent was obtained from the individual(s) for the publication of any potentially identifiable images or data included in this article.

## Author contributions

CR: Conceptualization, Investigation, Writing – original draft. EM: Data curation, Investigation, Writing – original draft, Conceptualization. SP: Investigation, Investigation, Data curation, Writing – original draft. SD: Data curation, Methodology, Writing – review & editing. MM: Data curation, Methodology, Writing – review & editing. LT: Formal analysis, Methodology, Supervision, Writing – review & editing. GZ: Formal analysis, Methodology, Supervision, Writing – review & editing. MP: Supervision, Writing – review & editing. MS: Writing – review & editing, Supervision. SR: Writing – review & editing, Supervision. GR: Conceptualization, Data curation, Writing – review & editing, Investigation, Supervision.
